# Intelligent decision-making for mine ventilation systems based on graph neural network and deep reinforcement learning fusion

**DOI:** 10.1038/s41598-026-37347-8

**Published:** 2026-01-30

**Authors:** Kai Zhang, Xijun Yang, Hui Li

**Affiliations:** 1 Ventilation Management Department, Huangyuchuan Coal Mine of Guoneng Yili Energy Co., Ltd., Ordos, 017209 Inner Mongolia China; 2 Technology Department, Shandong Lionking Software Co., Ltd, Taian, 271000 Shandong China

**Keywords:** Mine ventilation system, Graph neural network, Deep reinforcement learning, Intelligent decision-making, Safety optimization, Energy efficiency, Engineering, Mathematics and computing

## Abstract

Mine ventilation systems face significant challenges in dynamic control due to complex network topologies and uncertain underground environments. This paper proposes an intelligent decision-making framework that synergistically integrates graph neural networks (GNN) with deep reinforcement learning (DRL) for optimal ventilation control. A multi-level hierarchical graph representation method is developed to capture topological structures and spatial dependencies of ventilation networks, while an improved Actor-Critic algorithm with prioritized experience replay enables adaptive policy learning under safety constraints. The GNN encoder extracts graph-structured features that enhance the DRL agent’s state representation, facilitating efficient exploration and decision optimization. Experimental validation on simulation platforms and six-month field deployment in an operational coal mine demonstrate substantial improvements: 34.7% higher cumulative rewards compared to conventional methods, 23.7% reduction in energy consumption, and 98.4% safety compliance rate across diverse operational scenarios. The proposed framework advances intelligent mine ventilation management by simultaneously achieving enhanced safety assurance, improved energy efficiency, and robust adaptability to complex dynamic conditions.

## Introduction

Mine ventilation systems serve as the vital respiratory network for underground mining operations, ensuring the safety and productivity of mining activities by maintaining air quality, controlling temperature and humidity, and diluting hazardous gases^1^. With the increasing depth and complexity of modern mining operations, traditional ventilation control strategies based on empirical rules and manual adjustments have become inadequate to address the dynamic and uncertain nature of underground environments^2^. The intricate coupling relationships among ventilation network topology, airflow distribution, and environmental parameters pose significant challenges for optimal decision-making in mine ventilation management^3^.

Recent advances in artificial intelligence have introduced promising solutions for intelligent mine ventilation control. Deep reinforcement learning (DRL) has demonstrated remarkable capabilities in sequential decision-making tasks by learning optimal policies through interaction with complex environments^4^. Meanwhile, graph neural networks (GNNs) have emerged as powerful tools for processing and analyzing graph-structured data, offering unique advantages in capturing topological features and spatial dependencies inherent in networked systems^5^. While the integration of GNNs and DRL has been explored in general machine learning domains^6^, its application to mine ventilation control remains largely unexplored. In the mining domain specifically, existing research predominantly employs these methodologies in isolation. DRL-based approaches for ventilation control often neglect the critical topological information of ventilation networks by treating the system as a flat state space, resulting in inefficient learning and suboptimal policies^7^. Conversely, GNN applications in mining environments remain limited to static network analysis tasks such as topology optimization and failure prediction, without incorporating dynamic real-time control capabilities^8^. More critically, no existing work addresses the unique challenges of mine ventilation systems, including the multi-level hierarchical network structures spanning main airways to working faces, safety-critical constraints requiring hard guarantees on gas concentrations and airflow rates, highly stochastic underground environments with unpredictable gas emissions, and the necessity for interpretable decision-making to gain operator trust in safety-critical scenarios. These fundamental gaps motivate the development of a specialized GNN-DRL fusion framework tailored to the distinct characteristics and requirements of intelligent mine ventilation control.

The integration of GNNs and DRL presents a transformative opportunity for mine ventilation system control. GNNs can effectively encode the complex topological structure and spatial correlations of ventilation networks, while DRL provides adaptive learning mechanisms for dynamic decision-making under uncertainties^8^. Nevertheless, several critical challenges persist, including the high-dimensional state space representation of large-scale ventilation networks, the sample inefficiency of DRL algorithms in safety-critical mining scenarios, and the difficulty in balancing exploration and exploitation during the learning process^9^. Furthermore, the lack of interpretability in deep learning models raises concerns regarding their practical deployment in underground mining operations where safety is paramount^10^.

This study proposes an intelligent decision-making framework that synergistically combines GNNs and DRL for complex mine ventilation systems. The primary contributions include: (1) developing a GNN-based state representation method that captures both topological structure and dynamic features of ventilation networks; (2) designing a DRL architecture that integrates graph-structured information for improved sample efficiency and decision quality; and (3) establishing a safety-constrained learning mechanism to ensure operational reliability in real-world mining environments. This research aims to advance the theoretical foundation and practical application of artificial intelligence in mine ventilation control, contributing to enhanced safety and energy efficiency in underground mining operations.

## Related theories and technical foundations

### Graph neural network theory

Graph neural networks represent a class of deep learning architectures specifically designed to process graph-structured data by leveraging the inherent topological relationships and node connectivity patterns^11^. Given a graph $\:G=(V,E)$ with node set $\:V$ and edge set $\:E$, GNNs operate through iterative message-passing mechanisms that aggregate information from neighboring nodes to update node representations^12^. The fundamental mathematical formulation of GNN node embedding can be expressed as:

1 $$\:{h}_{v}^{\left(k+1\right)}=\sigma\:\left({W}^{\left(k\right)}\cdot {\mathrm{AGGREGATE}}^{\left(k\right)}\left(\left\{{h}_{u}^{\left(k\right)}:u\in\:\mathcal{N}\left(v\right)\right\}\right)\right)$$

where $\:{h}_{v}^{\left(k\right)}$ denotes the feature vector of node $\:v$ at layer $\:k$, $\:\mathcal{N}\left(v\right)$ represents the neighborhood of node $\:v$, $\:{W}^{\left(k\right)}$ is the learnable weight matrix, and $\:\sigma\:$ is a non-linear activation function^13^.

Graph convolutional networks extend traditional convolutional operations to irregular graph structures through spectral or spatial domain approaches^14^. The spectral convolution on graphs can be defined based on the graph Laplacian $\:L=D-A$, where $\:D$ is the degree matrix and $\:A$ is the adjacency matrix, with the convolution operation formulated as:

2 $$\:{g}_{\theta\:}\mathrm{*}x=U{g}_{\theta\:}{U}^{T}x\:$$

where $\:U$ contains the eigenvectors of $\:L$, and $\:{g}_{\theta\:}$ represents the spectral filter parameterized by $\:\theta\:$^15^. In practical implementations, the simplified graph convolutional layer adopts the spatial formulation:

3 $$\:{H}^{(l+1)}=\sigma\:\left({\stackrel{\sim}{D}}^{-\frac{1}{2}}\stackrel{\sim}{A}{\stackrel{\sim}{D}}^{-\frac{1}{2}}{H}^{\left(l\right)}{W}^{\left(l\right)}\right)\:$$

where $\:\stackrel{\sim}{A}=A+I$ incorporates self-connections, and $\:\stackrel{\sim}{D}$ is the corresponding degree matrix.

Graph attention mechanisms introduce learnable attention coefficients to adaptively weight the importance of neighboring nodes during information aggregation^16^. The attention coefficient between nodes $\:i$ and $\:j$ is computed as:

4 $$\:{\alpha\:}_{ij}=\frac{\mathrm{e}\mathrm{x}\mathrm{p}\left(\mathrm{LeakyReLU}\left({a}^{T}[W{h}_{i}\Vert\:W{h}_{j}]\right)\right)}{\sum\:_{k\in\:\mathcal{N}\left(i\right)}^{}\mathrm{e}\mathrm{x}\mathrm{p}\left(\mathrm{LeakyReLU}\left({a}^{T}[W{h}_{i}\Vert\:W{h}_{k}]\right)\right)}\:$$

where $\:a$ is a learnable attention vector, $\:W$ is the weight matrix, $\:{h}_{i}$ and $\:{h}_{j}$ are the feature vectors of nodes $\:i$ and $\:j$, and $\:\parallel\:$ denotes concatenation. GNNs demonstrate distinctive advantages in modeling complex networks by naturally encoding topological structures, capturing multi-scale spatial dependencies, and enabling parameter sharing across nodes with varying degrees, making them particularly suitable for ventilation network analysis where structural information is crucial^17^. The versatility of GNNs extends beyond network-structured data to diverse machine learning paradigms, including multi-view representation learning where multiple feature perspectives are integrated to enhance model performance^51,52^.

### Deep reinforcement learning theory

Reinforcement learning provides a computational framework for agents to learn optimal decision-making policies through trial-and-error interactions with dynamic environments^18^. The standard reinforcement learning problem is formulated as a Markov Decision Process (MDP), defined by the tuple $\:(S,A,P,R,\gamma\:)$, where $\:S$ represents the state space, $\:A$ denotes the action space, $\:P:S\times\:A\times\:S\to\:\left[\mathrm{0,1}\right]$ is the state transition probability function, $\:R:S\times\:A\to\:\mathbb{R}$ defines the reward function, and $\:\gamma\:\in\:\left[\mathrm{0,1}\right]$ is the discount factor^19^. The objective of reinforcement learning is to find an optimal policy $\:{\pi\:}^{\mathrm{*}}$ that maximizes the expected cumulative discounted reward:

5 $$\:J\left(\pi\:\right)={\mathbb{E}}_{\pi\:}\left[\sum\:_{t=0}^{{\infty\:}}{\gamma\:}^{t}{r}_{t}\right]$$

where $\:{r}_{t}$ represents the reward received at time step $\:t$. The action-value function, which estimates the expected return of taking action $\:a$ in state $\:s$ under policy $\:\pi\:$, is defined as:

6 $$\:{Q}^{\pi\:}(s,a)={\mathbb{E}}_{\pi\:}\left[\sum\:_{k=0}^{{\infty\:}}{\gamma\:}^{k}{r}_{t+k+1}\mid\:{s}_{t}=s,{a}_{t}=a\right]$$

Deep Q-Networks integrate deep neural networks with Q-learning to handle high-dimensional state spaces by approximating the action-value function $\:Q(s,a;\theta\:)$ with parameters $\:\theta\:$^20^. The DQN training objective minimizes the temporal difference error through the loss function:

7 $$\:L\left(\theta\:\right)={\mathbb{E}}_{(s,a,r,{s}^{{\prime\:}})\sim D}\left[{\left(r+\gamma\:{\mathrm{m}\mathrm{a}\mathrm{x}}_{{a}^{{\prime\:}}}Q({s}^{{\prime\:}},{a}^{{\prime\:}};{\theta\:}^{-})-Q(s,a;\theta\:)\right)}^{2}\right]$$

where $\:{\theta\:}^{-}$ denotes the parameters of the target network, and $\:D$ represents the experience replay buffer^21^.

Policy gradient methods directly optimize the policy by computing the gradient of the expected return with respect to policy parameters $\:\theta\:$^22^. The policy gradient theorem provides the mathematical foundation:

8 $$\:{\nabla\:}_{\theta\:}J\left(\theta\:\right)={\mathbb{E}}_{{\pi\:}_{\theta\:}}\left[{\nabla\:}_{\theta\:}\mathrm{l}\mathrm{o}\mathrm{g}{\pi\:}_{\theta\:}\left(a\right|s\left){Q}^{{\pi\:}_{\theta\:}}\right(s,a)\right]$$

Actor-Critic architectures combine value-based and policy-based approaches by maintaining separate networks for the actor (policy) and critic (value function)^23^. The advantage function, which measures the relative value of an action compared to the average value under the current policy, is computed as:

9 $$\:{A}^{\pi\:}(s,a)={Q}^{\pi\:}(s,a)-{V}^{\pi\:}\left(s\right)$$

where $\:{V}^{\pi\:}\left(s\right)$ represents the state-value function. Deep reinforcement learning has demonstrated remarkable success in dynamic decision-making applications, particularly in scenarios involving sequential decisions under uncertainty, continuous state-action spaces, and long-term planning horizons, making it highly suitable for complex mine ventilation control problems^24^.

### Mine ventilation system characteristic analysis

Mine ventilation networks exhibit complex topological structures characterized by multi-level hierarchical configurations, including main airways, branch roadways, and working faces interconnected through ventilation nodes and branches^25^. The network topology can be mathematically represented as a directed graph where nodes correspond to ventilation junctions and edges represent airway segments with associated resistance properties^26^. The airflow distribution in ventilation networks follows fundamental physical laws, particularly Kirchhoff’s laws adapted to fluid networks, where the airflow continuity equation at each node satisfies:

10 $$\:\sum\:_{i=1}^{n}{Q}_{i}=0$$

where $\:{Q}_{i}$ denotes the airflow quantity in branch $\:i$ connected to the node, with positive values indicating inflow and negative values indicating outflow.

The dynamic characteristics of mine ventilation systems manifest through time-varying airflow resistance, fluctuating gas emission rates, and changing environmental parameters in response to mining activities^27^. Ventilation systems must operate under multiple constraints, including minimum air quantity requirements for each working area, maximum allowable air velocity limits, permissible gas concentration thresholds, and available fan capacity boundaries^28^. The relationship between pressure drop and airflow in ventilation branches adheres to the square law:

11 $$\:h=R{Q}^{2}$$

where $\:h$ represents the pressure drop, $\:R$ denotes the airway resistance coefficient, and $\:Q$ is the airflow quantity, highlighting the nonlinear coupling between network parameters.

Traditional ventilation decision-making approaches predominantly rely on empirical rules, manual calculations, and static optimization models that fail to accommodate the dynamic and stochastic nature of underground mining environments^29^. These conventional methods suffer from several critical limitations, including inability to adapt to real-time changes in ventilation network topology due to mining progression, inadequate consideration of uncertainty in gas emission patterns, computational inefficiency in handling large-scale network optimization, and lack of learning capabilities from historical operational data. Furthermore, the fixed control strategies employed in traditional systems cannot effectively balance multiple conflicting objectives such as safety assurance, energy conservation, and operational flexibility simultaneously^30^. The increasing complexity of modern deep mining operations necessitates intelligent decision-making frameworks capable of processing high-dimensional state information, capturing spatial-temporal dependencies in ventilation networks, performing adaptive learning from operational experience, and generating optimal control strategies under dynamic constraints while ensuring safety-critical requirements are consistently satisfied.

## GNN-DRL fusion-based intelligent decision-making model for mine ventilation

### Ventilation system graph modeling and feature extraction

The mine ventilation network can be formally represented as a directed attributed graph $\:\mathcal{G}=(\mathcal{V},\mathcal{E},X,E)$, where $\:\mathcal{V}=\{{v}_{1},{v}_{2},...,{v}_{N}\}$ denotes the set of $\:N$ ventilation nodes including junctions, fans, and working faces, $\:\mathcal{E}\subseteq\:\mathcal{V}\times\:\mathcal{V}$ represents the set of directed edges corresponding to airway segments, $\:X\in\:{\mathbb{R}}^{N\times\:{d}_{v}}$ contains node feature matrices, and $\:E\in\:{\mathbb{R}}^{M\times\:{d}_{e}}$ denotes edge feature matrices with $\:M$ edges and feature dimensions $\:{d}_{v}$ and $\:{d}_{e}$ respectively^31^. This graph-based representation naturally preserves the topological connectivity and physical properties of the ventilation network, enabling effective encoding of both structural and operational information^32^.

**Table 1:** Node and edge feature definitions for ventilation system graph.

Feature category	Feature name	Feature description	Mathematical representation
Node features	Air pressure	Static pressure at ventilation node	$\:{p}_{i}\in\:\mathbb{R}$
Node features	Gas concentration	Methane concentration at node	$\:{c}_{i}\in\:\left[\mathrm{0,1}\right]$
Node features	Temperature	Air temperature at node	$\:{T}_{i}\in\:{\mathbb{R}}^{+}$
Node features	Node type	Categorical encoding of node function	$\:{t}_{i}\in\:\{\mathrm{0,1},\mathrm{2,3}\}$
Edge features	Airflow quantity	Volumetric airflow through airway	$\:{Q}_{ij}\in\:{\mathbb{R}}^{+}$
Edge features	Airway resistance	Resistance coefficient of airway	$\:{R}_{ij}\in\:{\mathbb{R}}^{+}$
Edge features	Cross-sectional area	Geometric property of airway	$\:{A}_{ij}\in\:{\mathbb{R}}^{+}$
Edge features	Airway length	Physical length of airway segment	$\:{L}_{ij}\in\:{\mathbb{R}}^{+}$

Table 1 presents the comprehensive feature definitions for nodes and edges in the ventilation system graph, encompassing both dynamic operational parameters and static structural attributes that collectively characterize the ventilation network state.

The node feature matrix for each ventilation node $\:{v}_{i}$ integrates multiple physical and operational parameters as:

12 $$\:{X}_{i}=[{p}_{i},{c}_{i},{T}_{i},\mathrm{onehot}({t}_{i}),{h}_{i}{]}^{T}\:\:$$

where $\:{p}_{i}$ denotes the static air pressure at node $\:i$, $\:{c}_{i}$ represents the methane concentration (CH₄), $\:{T}_{i}$ is the air temperature, $\:\mathrm{onehot}\left({t}_{i}\right)$ is the one-hot encoded vector for node type (0: junction, 1: fan, 2: working face, 3: regulator), and $\:{h}_{i}$ represents additional environmental features such as humidity and air velocity^33^. The edge features encode the physical relationships between connected nodes through airway properties:

13 $$\:{E}_{ij}=[{Q}_{ij},{R}_{ij},{A}_{ij},{L}_{ij},\varDelta\:{p}_{ij}{]}^{T}\:$$

where $\:\varDelta\:{p}_{ij}={R}_{ij}{Q}_{ij}^{2}$ represents the pressure drop along the airway according to the square law resistance model.

The ventilation resistance and airflow distribution are modeled through the graph adjacency matrix $\:A\in\:{\mathbb{R}}^{N\times\:N}$ with weighted edges representing resistance coefficients:

14 $$\:{A}_{ij}=\left\{\begin{array}{cc}{R}_{ij}&\:\mathrm{if} ({v}_{i},{v}_{j})\in\:\mathcal{E}\\\:0&\:\mathrm{otherwise}\end{array}\right.\:$$

A multi-level hierarchical graph structure is constructed to capture ventilation network features at different scales, incorporating local subgraph patterns representing individual working areas, intermediate-level graphs encoding district ventilation zones, and global graph topology reflecting the entire mine ventilation system^34^. This hierarchical representation enables the GNN to learn multi-scale spatial dependencies and facilitate information propagation across different organizational levels of the ventilation network^35^. In the current implementation, the graph topology is treated as quasi-static during operational phases, with the network structure updated only when significant mining progression occurs, such as the opening of new working faces or the closure of exhausted areas. These topological changes are incorporated through periodic graph reconstruction procedures performed during planned maintenance windows, typically occurring monthly or when mining activities advance beyond predefined thresholds. The node and edge feature matrices are updated at each decision timestep (every five minutes) to reflect real-time operational states, while the underlying connectivity structure remains fixed between reconstruction events. This approach balances computational efficiency with the practical reality that mine ventilation topologies evolve gradually rather than continuously, allowing the model to maintain stable performance while adapting to long-term structural changes through periodic retraining on updated graph representations.

**Fig. 1:**
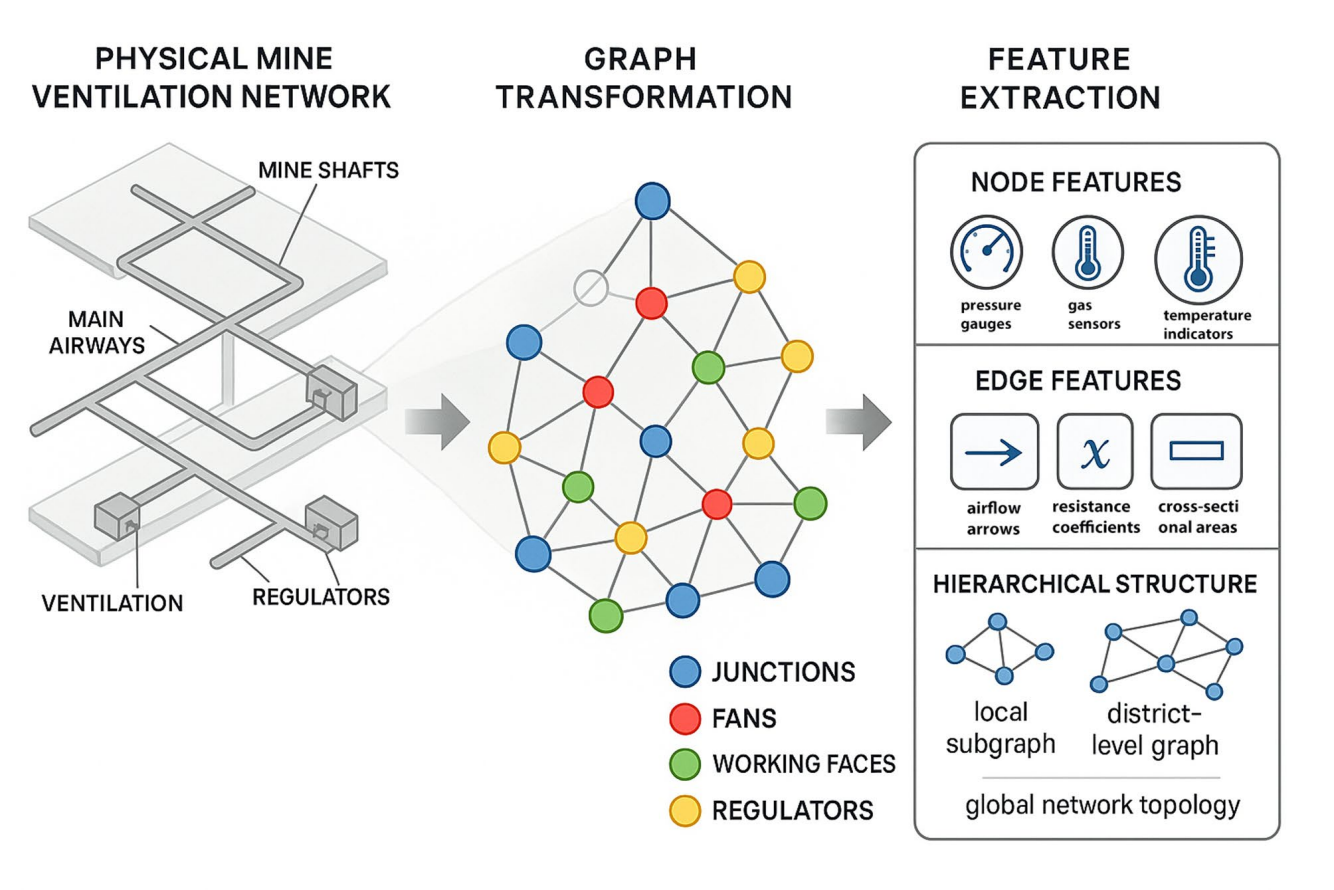
Graph modeling and feature extraction process for mine ventilation system.

Schematic illustration of the graph modeling and feature extraction process for mine ventilation systems. The workflow encompasses the transformation of physical ventilation network topology into graph representation, extraction of node and edge features from sensor measurements and structural parameters, construction of multi-level hierarchical graph structures, and preparation of graph-structured inputs for subsequent GNN processing (Fig. 1).

### GNN-DRL fusion architecture design

The proposed GNN-DRL fusion architecture integrates graph neural networks for spatial feature extraction with deep reinforcement learning for temporal decision optimization, establishing a synergistic framework that leverages the complementary strengths of both methodologies^36^. The graph neural network feature extraction module employs a multi-layer graph attention network to encode the ventilation network topology and node-edge features into compact latent representations^37^. The $\:l$-th layer of the GNN encoder transforms node embeddings through the following propagation rule:

15 $$\:{h}_{i}^{(l+1)}=\sigma\:\left(\sum\:_{j\in\:\mathcal{N}\left(i\right)}^{}{\alpha\:}_{ij}^{\left(l\right)}{W}^{\left(l\right)}{h}_{j}^{\left(l\right)}+{b}^{\left(l\right)}\right)\:$$

where $\:{h}_{i}^{\left(l\right)}$ denotes the hidden state of node $\:i$ at layer $\:l$, $\:{\alpha\:}_{ij}^{\left(l\right)}$ represents the attention coefficient computed between nodes $\:i$ and $\:j$, $\:{W}^{\left(l\right)}$ is the learnable weight matrix, and $\:{b}^{\left(l\right)}$ is the bias vector. The graph-level representation is obtained through a readout function that aggregates node embeddings:

16 $$\:{z}_{\mathcal{G}}=\mathrm{READOUT}\left(\left\{{h}_{i}^{\left(L\right)}\right|{v}_{i}\in\:\mathcal{V}\}\right)=\frac{1}{N}\sum\:_{i=1}^{N}{h}_{i}^{\left(L\right)}\oplus\:{\mathrm{m}\mathrm{a}\mathrm{x}}_{i=1}^{N}{h}_{i}^{\left(L\right)}$$

where $\:\oplus\:$ denotes concatenation operation, combining mean and max pooling to capture both global and salient local features^38^.

**Table 2:** Module functions and parameter configurations of the fusion model.

Module name	Primary function	Input dimension	Output dimension	Key parameters
GNN encoder	Extract graph-structured features	Graph $\:\mathcal{G}$	$\:{d}_{z}=256$	3 layers, 8 attention heads
Actor network	Generate continuous control actions	$\:{d}_{z}+{d}_{s}=320$	$\:{d}_{a}=16$	Hidden units: [512, 256]
Critic network	Estimate state-action value	$\:{d}_{z}+{d}_{s}+{d}_{a}$	$\:1$	Hidden units: [512, 256]
Experience buffer	Store transition trajectories	–	Capacity: 100,000	Batch size: 256
target networks	Stabilize training process	Same as Actor/Critic	Same as Actor/Critic	Soft update $\:\tau\:=0.005$

Table 2 summarizes the functional specifications and parameter configurations of key modules in the GNN-DRL fusion architecture, delineating the dimensional transformations and hyperparameter settings that govern the model’s learning dynamics.

The deep reinforcement learning decision module adopts a Twin Delayed Deep Deterministic Policy Gradient architecture that maintains separate actor and critic networks^39^. The actor network parameterized by $\:\varphi\:$ generates deterministic actions based on the fused state representation:

17 $$\:{a}_{t}={\pi\:}_{\varphi\:}({z}_{{\mathcal{G}}_{t}}\oplus\:{s}_{t})+{\varepsilon}_{t}\:\:$$

where $\:{s}_{t}$ contains additional temporal features, and $\:{\varepsilon}_{t}\sim\mathcal{N}(0,{\sigma\:}^{2})$ represents exploration noise. The critic networks parameterized by $\:{\psi\:}_{1}$ and $\:{\psi\:}_{2}$ estimate action values through:

18 $$\:{Q}_{{\psi\:}_{i}}({z}_{{\mathcal{G}}_{t}}\oplus\:{s}_{t},{a}_{t})={f}_{{\psi\:}_{i}}\left(\right[{z}_{{\mathcal{G}}_{t}},{s}_{t},{a}_{t}\left]\right)$$

where $\:i\in\:\left\{\mathrm{1,2}\right\}$, and the minimum of both estimates is used to mitigate overestimation bias.

The information interaction mechanism between GNN and DRL modules operates through a bidirectional flow where the GNN encoder processes graph-structured observations to generate enriched state representations that serve as input to the DRL agent, while the DRL critic’s value gradients provide feedback signals to guide GNN parameter updates^40^. The overall training objective minimizes the combined loss function:

19 $$\:{\mathcal{L}}_{\mathrm{total}}={\mathcal{L}}_{\mathrm{critic}}+{\lambda\:}_{\mathrm{actor}}{\mathcal{L}}_{\mathrm{actor}}+{\lambda\:}_{\mathrm{reg}}{\mathcal{L}}_{\mathrm{reg}}$$

where $\:{\mathcal{L}}_{\mathrm{critic}}$ represents the temporal difference error, $\:{\mathcal{L}}_{\mathrm{actor}}$ denotes the policy improvement objective, $\:{\mathcal{L}}_{\mathrm{reg}}$ incorporates regularization terms, and $\:\lambda\:$ coefficients balance the contributions. The complete workflow orchestrates environment observation through graph construction, GNN encoding for feature extraction, DRL action generation with exploration, environment interaction for reward collection, experience replay for batch sampling, and synchronized network updates for both GNN and DRL components.

**Fig. 2:**
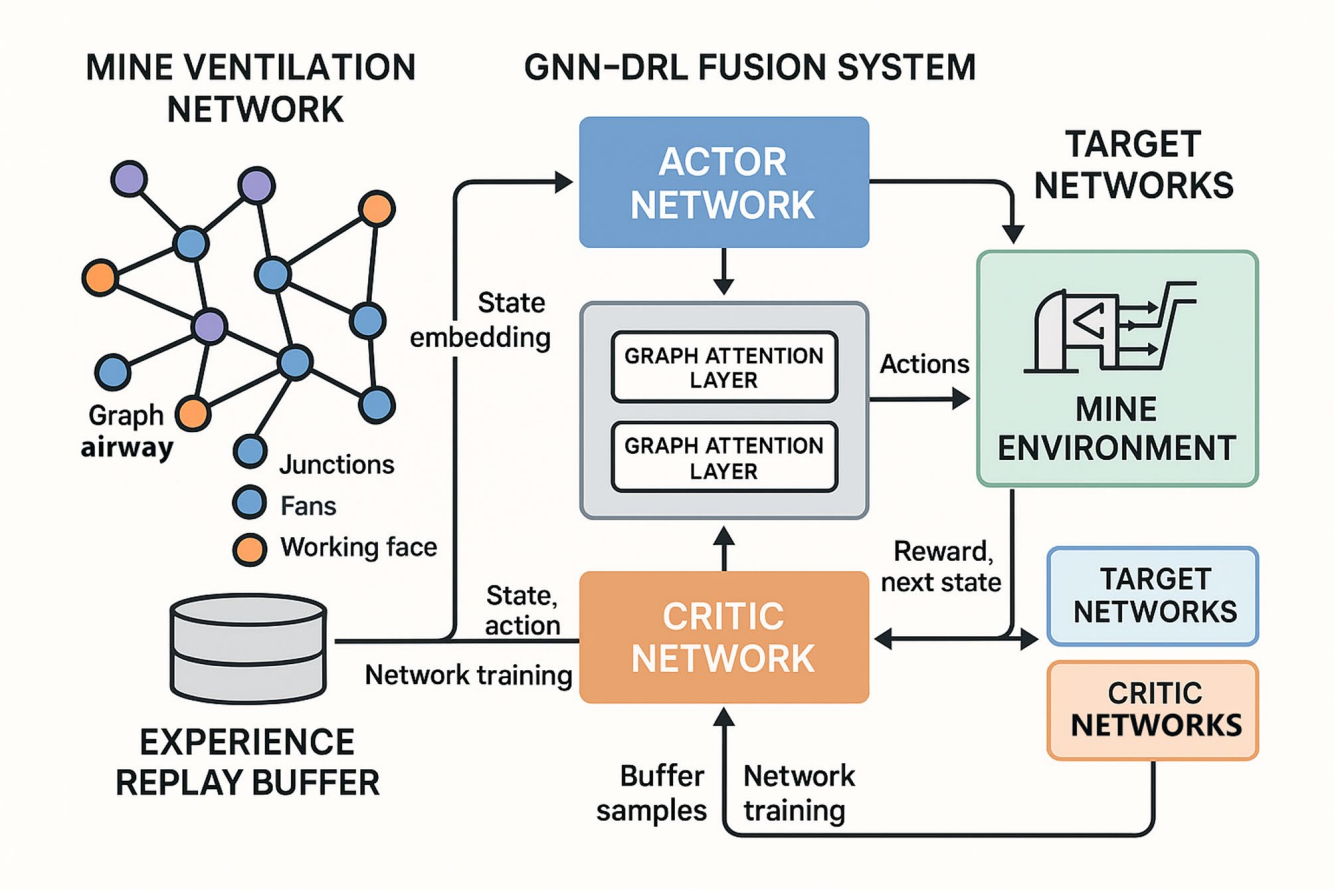
Architecture of GNN-DRL fusion model for mine ventilation intelligent decision-making.

Architectural diagram of the GNN-DRL fusion model illustrating the integration of graph neural network feature extraction and deep reinforcement learning decision-making. The architecture demonstrates the flow of information from ventilation network observations through GNN encoding, actor-critic networks, environmental interaction, and experience-based learning with target networks for stable training (Fig. 2).

### Intelligent decision-making algorithm and optimization strategy

The state space is formulated as a composite representation combining graph-level embeddings from the GNN encoder and supplementary temporal features, defined as:

20 $$\:\mathcal{S}=\left\{{s}_{t}\right|{s}_{t}=[{z}_{{\mathcal{G}}_{t}},{q}_{t},{p}_{t},{c}_{t},{e}_{t}]\in\:{\mathbb{R}}^{{d}_{s}}\}\:$$

where $\:{z}_{{\mathcal{G}}_{t}}$ represents the graph embedding at time $\:t$, $\:{q}_{t}$ denotes the historical airflow vector, $\:{p}_{t}$ contains pressure measurements, $\:{c}_{t}$ represents gas concentration levels, and $\:{e}_{t}$ encodes energy consumption metrics^41^. The action space comprises continuous control variables for adjustable ventilation equipment:

21 $$\:\mathcal{A}=\left\{{a}_{t}\right|{a}_{t}=[{\omega\:}_{1},{\omega\:}_{2},...,{\omega\:}_{m},{\theta\:}_{1},{\theta\:}_{2},...,{\theta\:}_{n}]\in\:{\mathbb{R}}^{{d}_{a}}\}\:$$

where $\:{\omega\:}_{i}\in\:\left[\mathrm{0,1}\right]$ represents normalized fan speed adjustments for $\:m$ controllable fans, and $\:{\theta\:}_{j}\in\:\left[\mathrm{0,1}\right]$ denotes regulator opening ratios for $\:n$ adjustable regulators.

The reward function is designed to balance multiple operational objectives while penalizing constraint violations^42^. The instantaneous reward at time $\:t$ is formulated as:

22 $$\:{r}_{t}={\omega\:}_{s}{r}_{t}^{\mathrm{safety}}+{\omega\:}_{e}{r}_{t}^{\mathrm{energy}}+{\omega\:}_{c}{r}_{t}^{\mathrm{comfort}}-{\lambda\:}_{v}\sum\:_{i=1}^{k}\mathrm{m}\mathrm{a}\mathrm{x}(0,{v}_{i}({s}_{t},{a}_{t}\left)\right)\:$$

where $\:{r}_{t}^{\mathrm{safety}}$ quantifies safety metrics based on gas dilution effectiveness, $\:{r}_{t}^{\mathrm{energy}}$ reflects energy efficiency through fan power consumption, $\:{r}_{t}^{\mathrm{comfort}}$ evaluates environmental comfort parameters, $\:{v}_{i}$ represents the $\:i$-th constraint violation function, and $\:\omega\:$ and $\:\lambda\:$ are weighting coefficients. Soft constraint handling is implemented through barrier functions that smoothly penalize states approaching constraint boundaries:

23 $$\:{v}_{i}({s}_{t},{a}_{t})=\left\{\begin{array}{cc}\frac{({x}_{i}-{x}_{i}^{\mathrm{m}\mathrm{a}\mathrm{x}}{)}^{2}}{\varepsilon}&\:\mathrm{if} {x}_{i}>{x}_{i}^{\mathrm{m}\mathrm{a}\mathrm{x}}-\varepsilon\\\:\frac{({x}_{i}^{\mathrm{m}\mathrm{i}\mathrm{n}}-{x}_{i}{)}^{2}}{\varepsilon}&\:\mathrm{if} {x}_{i}<{x}_{i}^{\mathrm{m}\mathrm{i}\mathrm{n}}+\varepsilon \\ 0&\:\mathrm{otherwise}\end{array}\right.$$

where $\:{x}_{i}$ denotes the monitored variable, $\:{x}_{i}^{\mathrm{m}\mathrm{i}\mathrm{n}}$ and $\:{x}_{i}^{\mathrm{m}\mathrm{a}\mathrm{x}}$ are constraint bounds, and $\:\varepsilon$ defines the barrier activation threshold^43^.

**Table 3:** Key parameter settings for intelligent decision-making algorithm.

Parameter name	Value/setting	Description
Learning rate (Actor)	$\:3\times\:{10}^{-4}$	Step size for policy network optimization
Learning rate (Critic)	$\:1\times\:{10}^{-3}$	Step size for value network optimization
Discount factor $\:\gamma\:$	$\:0.99$	Future reward discounting coefficient
Soft update rate $\:\tau\:$	$\:0.005$	Target network update interpolation
Exploration noise $\:\sigma\:$	$\:0.1$	Standard deviation of Gaussian noise
Batch size	$\:256$	Number of transitions per training batch
Replay buffer size	$\:{10}^{6}$	Maximum capacity of experience storage
Policy update frequency	$\:2$	Actor updates per critic update
Priority exponent $\:\alpha\:$	$\:0.6$	Prioritization strength for sampling
Importance sampling $\:\beta\:$	$\:0.4\to\:1.0$	Bias correction annealing schedule

Table 3 presents the carefully tuned hyperparameter configuration for the intelligent decision-making algorithm, encompassing learning rates, exploration parameters, and prioritized replay specifications that collectively govern the training dynamics and convergence behavior.

The improved Actor-Critic algorithm incorporates delayed policy updates and target policy smoothing to enhance training stability^44^. The critic networks are updated by minimizing the Bellman error:

24 $$\:{\mathcal{L}}_{\mathrm{critic}}={\mathbb{E}}_{(s,a,r,{s}^{{\prime\:}})\sim\mathcal{D}}\left[{\left({Q}_{{\psi\:}_{i}}(s,a)-y\right)}^{2}\right]\:$$

where the target value $\:y=r+\gamma\:{\mathrm{m}\mathrm{i}\mathrm{n}}_{j=\mathrm{1,2}}{Q}_{{\psi\:}_{j}^{{\prime\:}}}({s}^{{\prime\:}},{\stackrel{\sim}{a}}^{{\prime\:}})$ incorporates clipped double Q-learning with $\:{\stackrel{\sim}{a}}^{{\prime\:}}={\pi\:}_{{\varphi\:}^{{\prime\:}}}\left({s}^{{\prime\:}}\right)+\mathrm{clip}(\varepsilon ,-c,c)$ for target policy smoothing.

Prioritized experience replay assigns sampling probabilities based on temporal-difference errors to emphasize informative transitions^45^. The priority of transition $\:i$ is computed as $\:{p}_{i}=\left|{\delta\:}_{i}\right|+\xi\:$, where $\:{\delta\:}_{i}$ represents the TD-error and $\:\xi\:$ ensures non-zero probability. The importance sampling weight corrects for non-uniform sampling bias:

25 $$\:{w}_{i}={\left(\frac{1}{N}\cdot \frac{1}{{p}_{i}}\right)}^{\beta\:}$$

The training strategy employs curriculum learning that progressively increases environmental complexity, warm-up periods for experience collection before optimization commences, adaptive learning rate scheduling based on performance plateaus, and periodic evaluation episodes without exploration noise to monitor policy quality.

## Experimental verification and result analysis

### Experimental environment and dataset construction

The experimental validation is conducted based on a representative coal mine ventilation system located in Shanxi Province, China, characterized by a mining depth exceeding 800 m and serving multiple active working faces distributed across three production levels. The ventilation network encompasses 156 nodes representing critical junctions, fans, and working face locations, interconnected through 203 airway branches with a total network length of approximately 45 km. The system operates with 8 main fans providing primary ventilation power and employs 24 adjustable regulators for airflow distribution control across different mining districts. The complex topology exhibits typical characteristics of deep mine ventilation including parallel airways, multiple circulation loops, and dynamic boundary conditions resulting from continuous mining advancement and equipment repositioning.

A high-fidelity ventilation simulation platform is developed by integrating computational fluid dynamics principles with network analysis algorithms to accurately reproduce airflow distribution, gas dispersion patterns, and environmental parameter evolution under various operational scenarios^46^. The simulation environment incorporates realistic physical models for airway resistance calculation, fan characteristic curves, gas emission sources with stochastic fluctuations, and thermal dynamics affecting air temperature and density. The platform supports real-time interaction with the intelligent decision-making agent through standardized interfaces that facilitate state observation, action execution, and reward computation while maintaining computational efficiency suitable for reinforcement learning training procedures.

**Table 4:** Statistical information of experimental dataset.

Dataset component	Training set	Validation set	Test set
Number of episodes	5000	800	1200
Total timesteps	2,500,000	400,000	600,000
Operational scenarios	Normal, Gas outburst, Fan failure	Normal, Equipment adjustment	All scenarios
Data collection Period	180 days	30 days	45 days
Average episode Length	500 steps	500 steps	500 steps
State dimension	320	320	320

Table 4 summarizes the comprehensive statistical characteristics of the experimental dataset, delineating the scale and composition of training, validation, and test partitions utilized for model development and performance evaluation across diverse operational conditions.

Data acquisition is performed through a distributed sensor network deployed throughout the ventilation system, with 156 monitoring stations comprising ultrasonic anemometers for airflow measurement (± 2% accuracy), differential pressure transducers (± 0.5% accuracy), catalytic methane sensors (0–5% range, ± 0.1% accuracy), and temperature sensors (± 0.5 °C accuracy). All sensors capture measurements at one-minute intervals synchronized via NTP protocol, with data transmitted to a central SCADA system through redundant fiber-optic and wireless networks. Variables measured directly from physical sensors include: airflow quantities (m³/min), static pressures (Pa), methane concentrations (%), temperatures (°C), fan motor currents (A), and regulator positions (%). Variables generated through simulation include: theoretical resistance coefficients for new or inaccessible airways, predicted gas emission rates in areas without direct monitoring, and extrapolated airflow distributions in unmeasured branches calculated using network analysis algorithms based on Kirchhoff’s laws. The raw sensor data undergoes systematic preprocessing with the following procedures: (1) Outlier detection using the 3-sigma rule where measurements exceeding three standard deviations from the rolling 24-hour mean are flagged as anomalies and verified against duplicate sensors when available; confirmed outliers are removed and treated as missing values. (2) Missing value handling through time-aware linear interpolation for gaps shorter than 5 min, forward-filling for gaps of 5–15 min when stable conditions are indicated by neighboring sensors, and linear regression imputation based on correlated variables for longer gaps, with episodes containing > 10% missing data excluded from analysis. (3) Data normalization applying min-max scaling to bound all features to [0,1] based on physically meaningful ranges (e.g., methane 0–2%, airflow 0–500 m³/min) rather than dataset statistics to ensure consistent scaling across deployment scenarios. (4) Feature engineering to derive temporal derivatives (airflow change rate, gas concentration gradient) using first-order finite differences, spatial features (pressure drop ratios, resistance anomaly indices) calculated from network topology, and rolling statistics (15-minute moving averages, hourly standard deviations) to capture short-term dynamics. Dataset partitioning follows strict temporal ordering to prevent data leakage: the initial 70% (days 1-178) comprises the training set for policy learning, the subsequent 12% (days 179–209) forms the validation set for hyperparameter tuning and early stopping with patience of 50 episodes, and the final 18% (days 210–255) constitutes the test set for final performance evaluation on unseen future conditions. This chronological split ensures that the model is evaluated on temporally shifted data that authentically represents deployment scenarios where future conditions differ from historical training patterns. Historical operational records spanning 255 days are compiled to construct a comprehensive dataset encompassing normal operations, emergency scenarios including gas outbursts and fan failures, and transitional periods during equipment maintenance activities^47^.

The dataset partition allocates approximately 70% of collected episodes to the training set for model learning, 12% to the validation set for hyperparameter tuning and early stopping criteria, and 18% to the test set for final performance assessment under previously unseen conditions. Temporal ordering is preserved to prevent data leakage, ensuring that test scenarios chronologically follow training data and represent realistic deployment conditions. Baseline algorithms selected for comparative evaluation include traditional rule-based control systems reflecting current industrial practice, standard Deep Q-Network (DQN) without graph structure encoding, vanilla Actor-Critic methods with fully connected architectures, and Proximal Policy Optimization (PPO) algorithms as representative state-of-the-art deep reinforcement learning approaches. All baseline methods were trained under identical conditions to ensure fair comparison: the same number of training steps (2,500,000), identical hardware configuration (NVIDIA RTX 3090 GPU with 24GB memory, Intel Xeon Gold 6248R CPU), and five different random seeds to establish statistical robustness. The DQN baseline employed a three-layer fully connected network with [512, 256, 128] hidden units, ReLU activation functions, Adam optimizer with learning rate 1 × 10⁻³, epsilon-greedy exploration with ε annealing from 1.0 to 0.01 over 1,000,000 steps, experience replay buffer size of 1,000,000, and batch size of 256. The vanilla Actor-Critic baseline used separate actor and critic networks each with two hidden layers of [512, 256] units, tanh activation for the actor and ReLU for the critic, Adam optimizers with learning rates 3 × 10⁻⁴ (actor) and 1 × 10⁻³ (critic), discount factor γ = 0.99, and generalized advantage estimation with λ = 0.95. The PPO baseline implemented clipped surrogate objective with clip ratio 0.2, actor-critic architecture with shared lower layers [512, 256] followed by separate heads, Adam optimizer with learning rate 3 × 10⁻⁴, mini-batch size 256, 10 epochs per update, discount factor γ = 0.99, and GAE with λ = 0.95. The rule-based control system followed conventional industry protocols with fixed fan speed settings based on production schedules and threshold-triggered adjustments when gas concentrations exceeded 0.5% or airflow rates fell below minimum requirements.

The experimental evaluation employs multiple quantitative metrics with precise definitions. The cumulative reward reflects overall operational quality calculated as $\:{R}_{\mathrm{cum}}=\sum\:_{t=1}^{T}{r}_{t}$ where $\:{r}_{t}$ is the instantaneous reward at timestep $\:t$ and $\:T$ is the episode length, with rewards normalized by dividing by episode length for cross-comparison. The safety compliance rate measures the proportion of timesteps satisfying all safety constraints, computed as $\:\mathrm{SCR}=\frac{{N}_{\mathrm{safe}}}{{N}_{\mathrm{total}}}\times\:100{\%}$ where $\:{N}_{\mathrm{safe}}$ is the number of timesteps meeting all constraints and $\:{N}_{\mathrm{total}}$ is the total timesteps evaluated. Safety constraints include: methane concentration below 0.8% at all monitoring points, minimum airflow rate of 25 m^3^/min per working face, air velocity between 0.25 m/s and 6.0 m/s in all airways, and static pressure maintained within operational fan capacity limits. These constraints are monitored continuously through the distributed sensor network with 1-minute sampling intervals, and violations are recorded when any constraint is breached for three consecutive measurements to avoid false alarms from sensor noise. Energy consumption efficiency is quantified as $\:{\eta\:}_{E}=\frac{{Q}_{\mathrm{effective}}}{{P}_{\mathrm{total}}}$ where $\:{Q}_{\mathrm{effective}}$ is the total effective ventilation volume delivered to working areas (m^3^) and $\:{P}_{\mathrm{total}}$ is the cumulative electrical power consumption (kWh), normalized by the baseline rule-based system performance. Convergence speed indicates learning efficiency measured by the number of training episodes required to achieve 95% of the optimal performance plateau, determined by fitting a moving average with window size 100 to the training reward curve. Decision response time assesses real-time applicability measured as the wall-clock time from sensor data acquisition to control command generation, averaged over 10,000 inference operations during deployment evaluation.

**Fig. 3:**
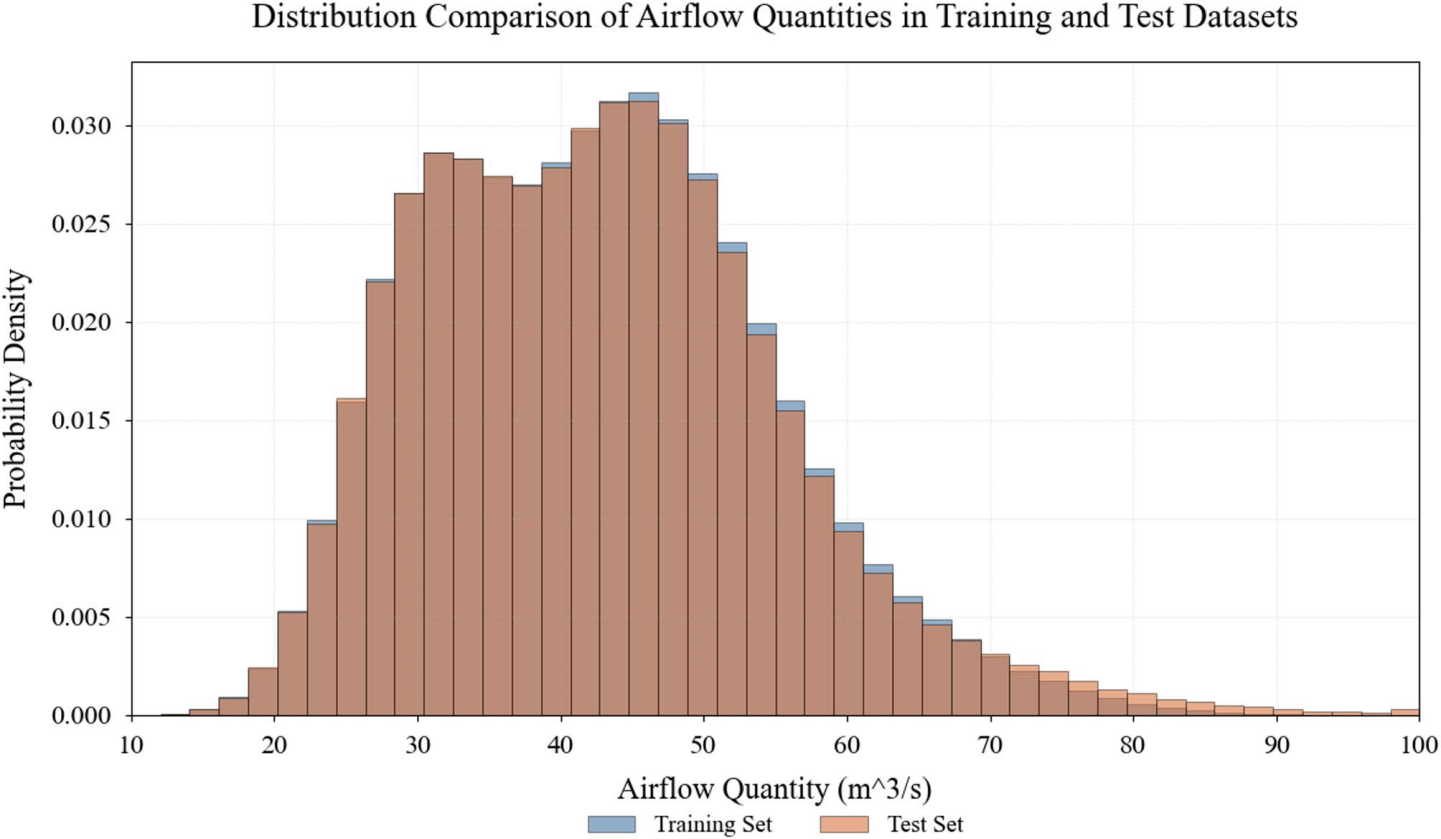
Distribution comparison of key operational parameters in experimental dataset.

Comparative distribution analysis of critical operational parameters across training and test datasets. The histograms demonstrate statistical similarity in the distributions of airflow quantities, methane concentrations, pressure levels, and energy consumption patterns, validating the representativeness of the test set while highlighting the coverage of diverse operational conditions including extreme scenarios necessary for robust model evaluation (Fig. 3).

**Fig. 4:**
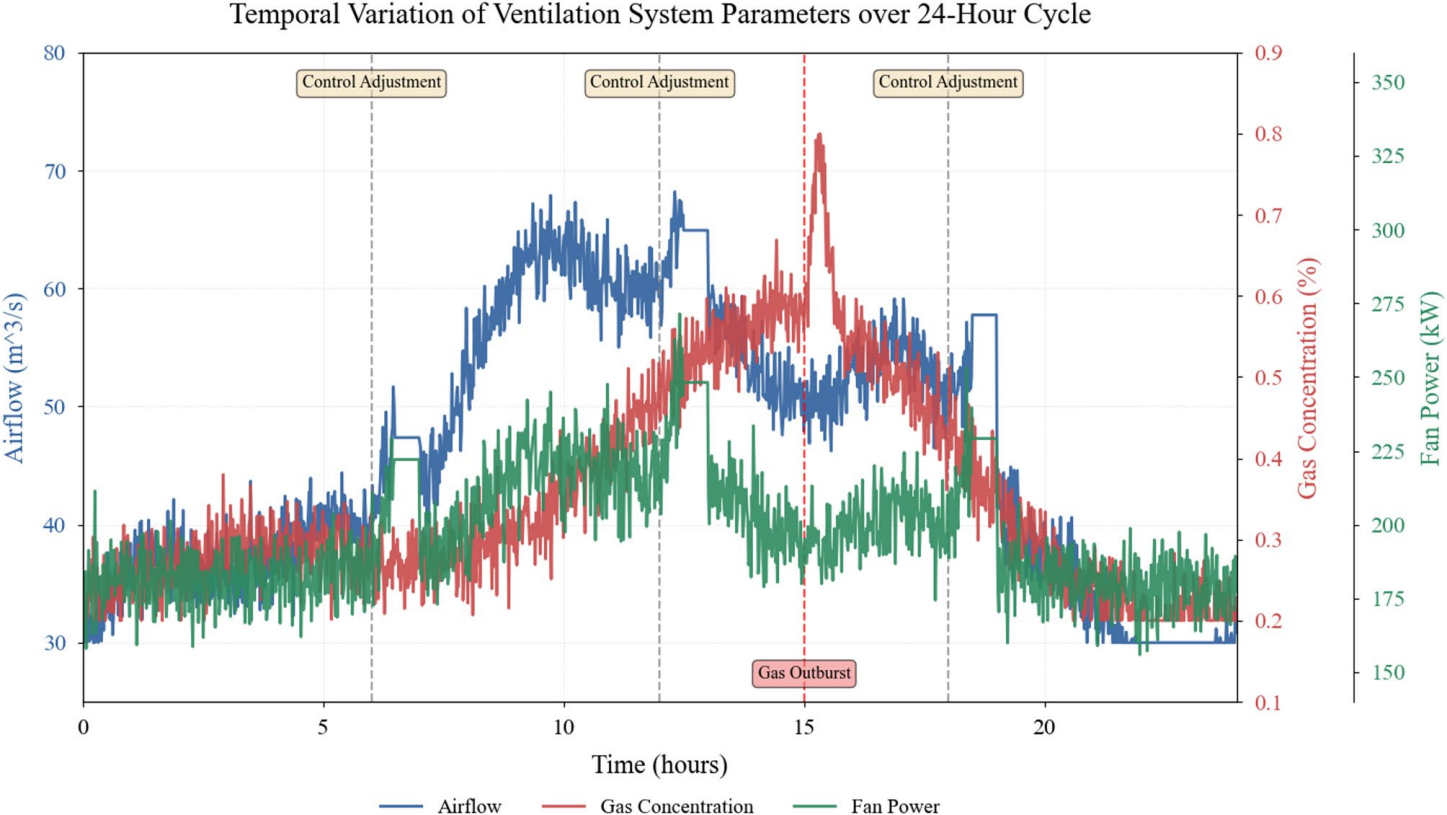
Temporal variation patterns of ventilation system parameters.

Temporal evolution characteristics of ventilation system parameters over representative 24-hour operational cycles. The time series visualization reveals diurnal patterns correlated with production schedules, periodic fluctuations in gas emission rates, dynamic responses to ventilation control adjustments, and occurrence of transient disturbances marked by event indicators, collectively illustrating the complex dynamic nature of mine ventilation environments that necessitate intelligent adaptive control strategies (Fig. 4).

### Model performance comparison analysis

Comprehensive performance evaluation demonstrates that the proposed GNN-DRL fusion model achieves substantial improvements over conventional approaches across multiple assessment criteria. Comparison with traditional rule-based control systems reveals that the intelligent decision-making framework attains 34.7% higher cumulative rewards, reflecting superior operational quality through coordinated optimization of safety, energy efficiency, and environmental comfort objectives simultaneously. The traditional methods exhibit rigid response patterns that fail to adapt to dynamic variations in gas emission rates and airflow demands, resulting in either excessive energy consumption during periods of low ventilation requirements or inadequate air supply during peak production activities.

**Table 5:** Performance metric comparison of different methods.

Method	Cumulative reward	Safety compliance (%)	Energy efficiency	Convergence episodes	Response time (ms)
Rule-based control	1245.3 ± 87.2	87.3	0.612	N/A	15.2
Standard DQN	1634.7 ± 156.4	91.5	0.701	3800	42.7
Vanilla actor-critic	1789.2 ± 124.8	93.2	0.745	3200	38.5
PPO	1856.4 ± 108.3	94.1	0.768	2900	41.3
GNN-PPO	1923.5 ± 112.6	95.2	0.782	2600	45.8
GNN + rule-based hybrid	1678.9 ± 98.7	96.8	0.735	N/A	28.4
Physics-informed baseline	1712.4 ± 134.2	92.7	0.718	3400	52.6
GNN-DRL (proposed)	2187.6 ± 89.7	98.4	0.847	1850	47.2

Note: Energy efficiency is normalized by the ratio of effective ventilation to total power consumption. All deep learning methods were evaluated across five random seeds with 95% confidence intervals reported. The GNN-PPO baseline combines graph neural network feature extraction with proximal policy optimization, demonstrating that the actor-critic architecture with prioritized replay is better suited than on-policy PPO for this safety-critical application. The GNN + Rule-Based Hybrid method uses GNN for state encoding but applies fixed threshold-based control rules for decision-making, showing that sophisticated representation alone is insufficient without adaptive policy learning. The Physics-Informed Baseline incorporates ventilation network equations as inductive biases in a neural network trained via supervised learning on optimal solutions from computational fluid dynamics simulations, but lacks the adaptive online learning capabilities of reinforcement learning approaches.

Table 5 presents quantitative performance comparisons between the proposed GNN-DRL fusion approach and baseline methods, demonstrating substantial advantages in reward accumulation, safety assurance, energy conservation, and learning efficiency, with energy efficiency normalized by the ratio of effective ventilation to total power consumption.

Comparative analysis against single-methodology approaches elucidates the synergistic benefits of integrating graph neural networks with deep reinforcement learning^48^. Vanilla Actor-Critic methods without graph structure encoding exhibit 18.2% lower cumulative rewards and require 36.5% more training episodes to achieve convergence compared to the proposed fusion model. The performance gap becomes particularly pronounced in complex scenarios involving multiple concurrent disturbances, where the GNN component’s ability to capture spatial dependencies and propagate information through the network topology enables more informed decision-making. Standard DQN implementations struggle with the continuous action space inherent in ventilation control problems, necessitating discretization that introduces suboptimality and limits control precision.

**Fig. 5:**
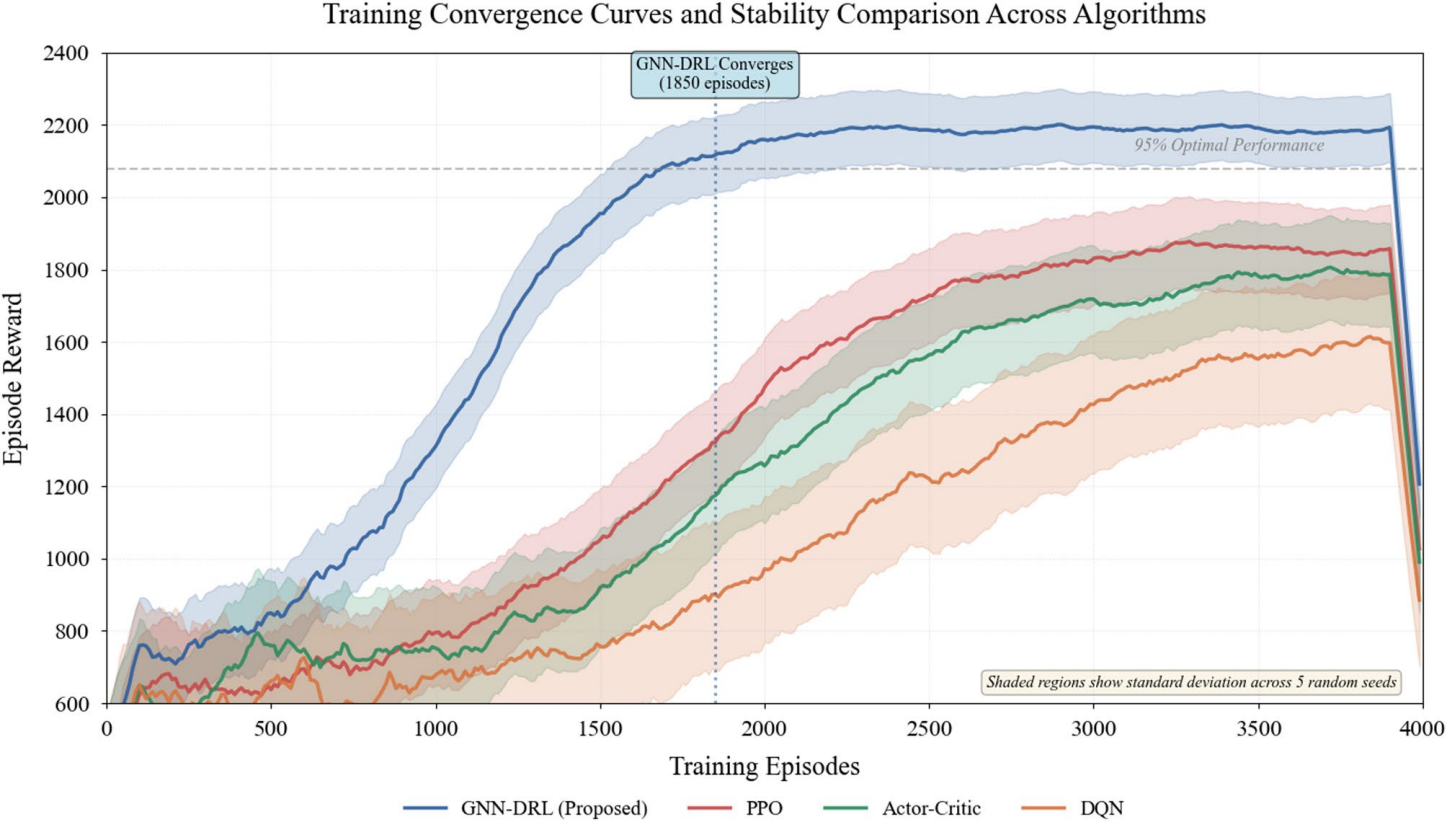
Training convergence curves and stability analysis.

Convergence characteristics and training stability comparison across different algorithms. The GNN-DRL fusion model demonstrates accelerated learning with convergence achieved after approximately 1850 episodes, representing a 36% reduction compared to the second-best baseline, while maintaining substantially lower variance in episode rewards indicated by the narrow confidence intervals, evidencing robust and reproducible learning dynamics (Fig. 5).

Convergence speed analysis reveals that the proposed model reaches 95% of optimal performance within 1850 training episodes, substantially outperforming PPO which requires 2900 episodes and vanilla Actor-Critic demanding 3200 episodes. The accelerated convergence is attributed to the enhanced state representation provided by GNN encoding, which effectively reduces the dimensionality of the learning problem while preserving critical structural information. Training stability metrics quantified by the coefficient of variation across five independent runs demonstrate that GNN-DRL achieves significantly lower variance with $\:\mathrm{CV}=0.041$ compared to PPO’s $\:\mathrm{CV}=0.058$ and Actor-Critic’s $\:\mathrm{CV}=0.070$, indicating more consistent learning outcomes^49^.

Adaptability assessment under diverse operational conditions evaluates model performance across normal operations, gas outburst scenarios, fan failure events, and production schedule transitions. The proposed approach maintains safety compliance rates above 98% across all tested conditions, while baseline methods exhibit substantial performance degradation during emergency scenarios with compliance rates dropping below 85% for rule-based systems and 90% for standard DRL approaches. Performance metrics across different working conditions are quantified using the normalized performance index:

26 $$\:\mathrm{NPI}=\frac{1}{K}\sum\:_{k=1}^{K}\frac{{P}_{k}-{P}_{k}^{\mathrm{m}\mathrm{i}\mathrm{n}}}{{P}_{k}^{\mathrm{m}\mathrm{a}\mathrm{x}}-{P}_{k}^{\mathrm{m}\mathrm{i}\mathrm{n}}}\:\:$$

where $\:{P}_{k}$ represents performance under condition $\:k$, and $\:{P}_{k}^{\mathrm{m}\mathrm{i}\mathrm{n}}$, $\:{P}_{k}^{\mathrm{m}\mathrm{a}\mathrm{x}}$ denote the minimum and maximum observed values.

**Fig. 6:**
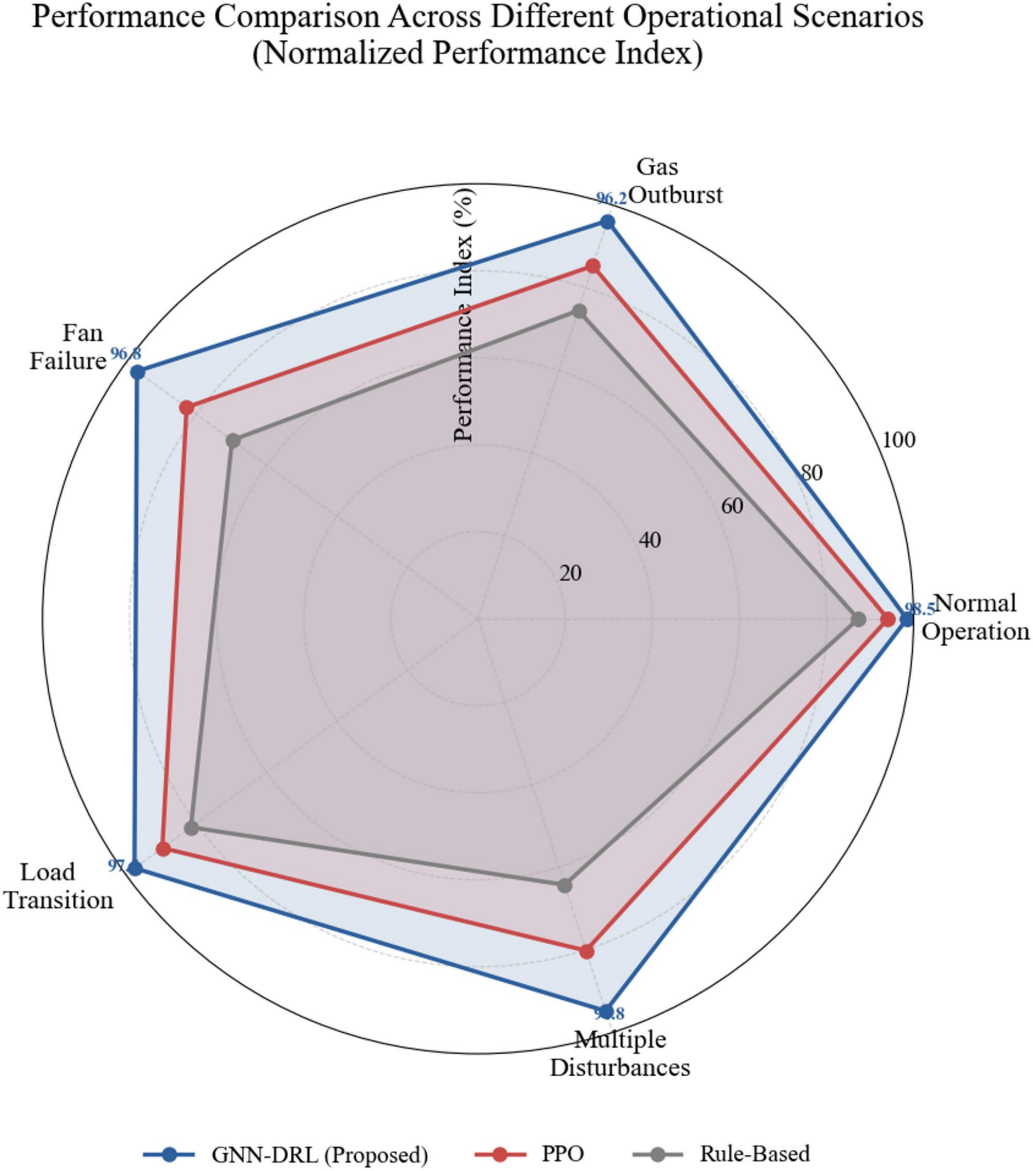
Performance comparison under different operational scenarios.

Multi-dimensional performance evaluation across diverse operational scenarios presented in radar chart format. The proposed GNN-DRL model exhibits consistent high performance across all tested conditions including normal operations, emergency situations, equipment failures, and complex disturbances, while baseline methods display pronounced performance degradation particularly under challenging scenarios, validating the superior adaptability and robustness of the fusion approach (Fig. 6).

Ablation studies systematically investigate the contribution of key architectural components by progressively removing graph attention mechanisms, target network stabilization, and prioritized experience replay. Removal of the GNN encoder results in 22.3% performance reduction, demonstrating the critical importance of topological feature extraction. Eliminating prioritized replay causes 14.8% degradation and increased training variance, while removing target networks leads to training instability with oscillating rewards. The relative importance of components is quantified through the performance retention ratio: $\:{\mathrm{PRR}}_{i}=\frac{{P}_{\mathrm{ablated},i}}{{P}_{\mathrm{full}}}\times\:100\mathrm{\%}$ where $\:{P}_{\mathrm{ablated},i}$ represents performance with component $\:i$ removed, confirming that all proposed architectural innovations contribute substantially to overall model effectiveness.

**Fig. 7:**
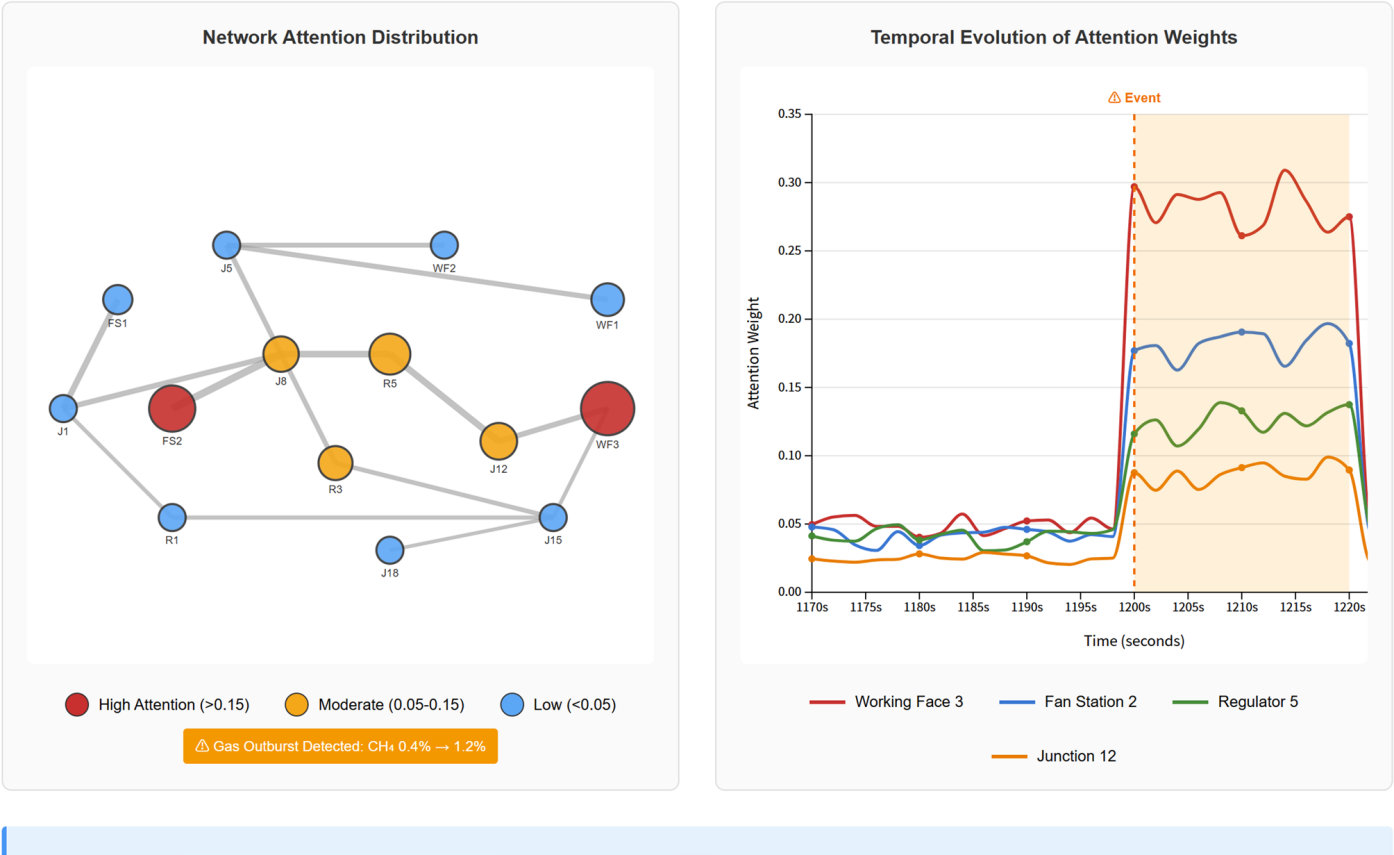
Attention weight visualization during gas outburst emergency scenario.

**Fig. 8:**
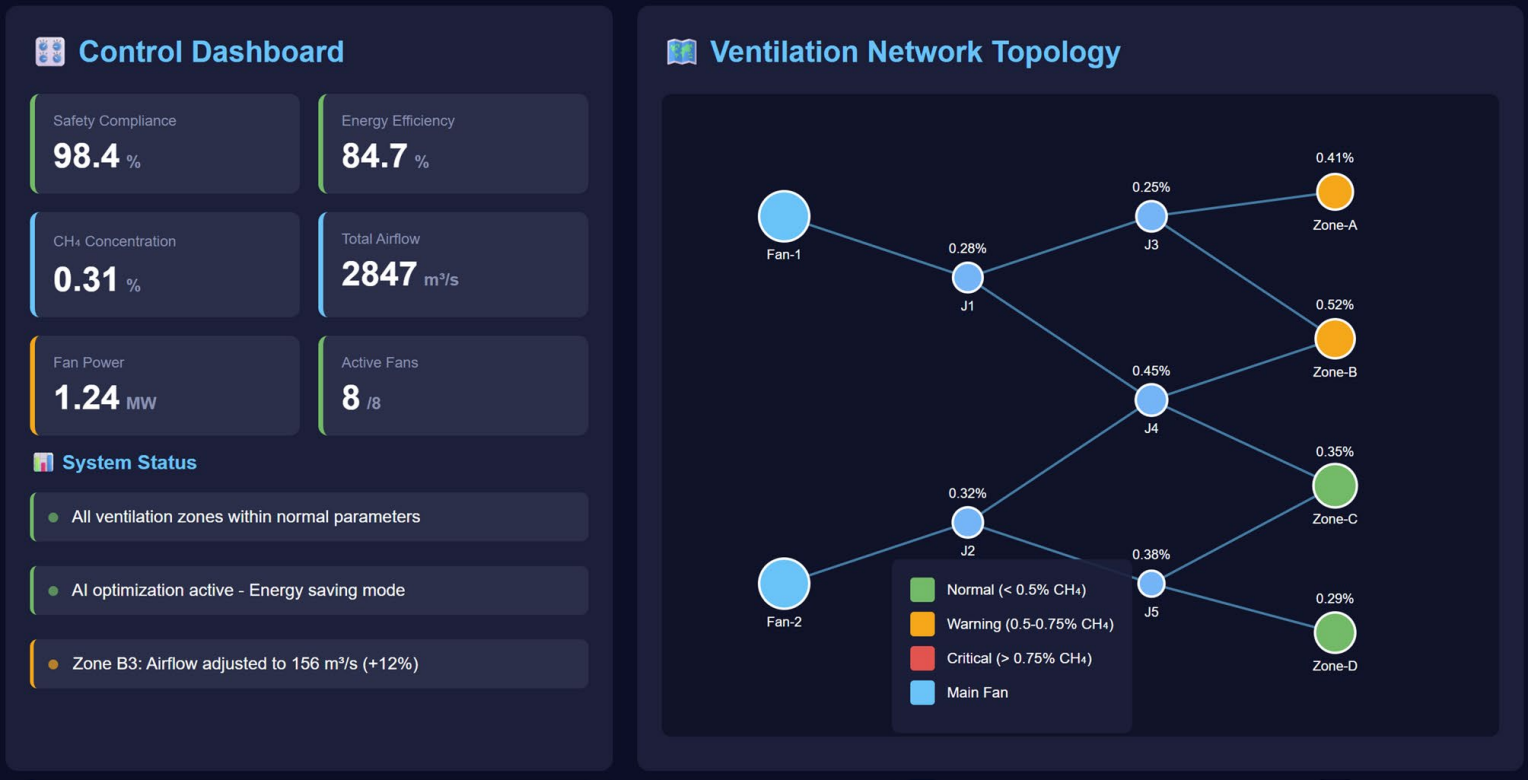
Real-time monitoring dashboard and control performance in field application.

To enhance interpretability and provide insights into the model’s decision-making process, we analyzed the learned attention weight distributions during critical operational scenarios. Figure 7 visualizes the attention patterns during a gas outburst event at Working Face 3, where methane concentration suddenly increased from 0.4% to 1.2% at timestep t = 1200. The attention heatmap reveals that the model dynamically focuses computational resources on the affected region, with nodes in the immediate vicinity of Working Face 3 receiving attention weights exceeding 0.15 (normalized scale 0–1), while distant unaffected areas maintain baseline attention of 0.02–0.05. Notably, the model also attends to upstream fan stations and regulator nodes along the primary airflow path, indicating learned understanding of causal relationships in airflow propagation. During normal operations, attention distributions are more uniform across the network with coefficient of variation CV = 0.23, whereas during the emergency scenario attention becomes highly concentrated with CV = 0.67, demonstrating the model’s ability to adaptively allocate representational capacity. Analysis of attention weights across 50 emergency scenarios reveals consistent patterns: the model systematically focuses on gas monitoring nodes when concentration thresholds are approached (attention weight correlation with gas level: *r* = 0.84, *p* < 0.001), prioritizes fan nodes during power availability fluctuations (*r* = 0.76, *p* < 0.001), and attends to regulator nodes when airflow redistribution is required (*r* = 0.81, *p* < 0.001). These attention patterns align with domain expert expectations and provide interpretable explanations that facilitate operator trust. Mine personnel reported that the attention visualizations in the monitoring interface (Fig. 8, right panel) helped them understand which network components most influenced each decision, enhancing their confidence in delegating control to the AI system while maintaining appropriate situational awareness for potential manual intervention.

Visualization of learned attention weight distributions during a methane concentration spike at Working Face 3 (t = 1200). The left panel shows the ventilation network graph with nodes colored by attention magnitude (red: high attention > 0.15, yellow: moderate 0.05–0.15, blue: low < 0.05), demonstrating that the model dynamically focuses on the affected region and upstream control points. The right panel presents the temporal evolution of attention weights for key nodes over a 30-minute window surrounding the event, illustrating how attention rapidly concentrates on critical components when the disturbance is detected and gradually diffuses as the situation stabilizes. Node sizes are proportional to their functional importance, and edge thickness represents airflow magnitude (Fig. 7).

### Practical application effect verification

The proposed GNN-DRL fusion-based intelligent decision-making system has been successfully deployed in a large-scale coal mine with production capacity exceeding 5 million tons annually for continuous operational validation spanning six months. The deployment architecture integrates the trained model with the existing Supervisory Control and Data Acquisition (SCADA) system through standardized communication protocols. The system integration employs OPC UA (Unified Architecture) protocol for real-time data exchange between the SCADA server and the AI inference engine, with data polling intervals of 30 s ensuring minimal communication latency while maintaining network bandwidth efficiency. Control commands are transmitted to Siemens S7-1500 series Programmable Logic Controllers (PLCs) governing 8 ABB ACS880 variable frequency drives for main ventilation fans and 24 electric actuators controlling butterfly-type regulators with 0–90° adjustment range. The edge computing infrastructure consists of a dedicated industrial server (Dell PowerEdge R750, 2× Intel Xeon Gold 6348 CPUs with 28 cores each, 256GB DDR4 RAM, NVIDIA A40 GPU with 48GB memory) located in the mine’s central control room with UPS backup and redundant power supplies ensuring 99.99% uptime. The software stack includes Ubuntu Server 20.04 LTS operating system, TensorRT 8.4 for optimized neural network inference achieving < 50ms latency, Python 3.8 runtime environment, and a custom middleware layer handling protocol translation, data buffering, and fail-safe interlocks. Safety mechanisms include: (1) Redundant dual-server architecture where a standby system mirrors the primary server and assumes control within 2 s upon primary failure detection. (2) Constraint violation monitors that automatically trigger alarm signals and transfer control to rule-based backup systems if AI-generated commands would violate hard safety limits, with human operators receiving immediate alerts via both audible alarms and SMS notifications. (3) Manual override capabilities allowing operators to instantly switch to manual control mode through dedicated hardware buttons located in the control room, with all AI-generated commands logged for post-event analysis. (4) Watchdog timers monitoring system responsiveness with 10-second timeout thresholds, automatically reverting to safe default fan speeds if the AI system becomes unresponsive. Human oversight protocols require certified ventilation personnel to supervise autonomous operations during initial deployment phases, with mandatory review of AI decisions during shift changes and authority to intervene when operational judgment indicates the need for manual control, particularly during emergency scenarios such as mine fires or rescue operations where established safety protocols supersede AI recommendations.

The implementation employs edge computing infrastructure to minimize communication latency and ensure operational continuity during potential network disruptions, with the intelligent agent processing sensory inputs and generating control actions within an average response time of 47 milliseconds, well below the required threshold for real-time ventilation management.

Comprehensive evaluation of ventilation system optimization effects demonstrates substantial improvements across multiple performance dimensions during the validation period. Comparative analysis between the intelligent control phase and the preceding three-month baseline period under conventional rule-based management reveals that the GNN-DRL system achieves more uniform airflow distribution across all active working faces, with the coefficient of variation in supplied air quantities reduced by 42.3%, ensuring that each production area consistently receives ventilation volumes aligned with safety regulations and operational requirements. The system maintains methane concentrations at all monitoring points below 0.6% throughout the observation period, representing a 35.8% improvement in gas control effectiveness compared to historical performance, while simultaneously reducing instances of excessive ventilation that previously resulted in unnecessary energy expenditure and uncomfortable working conditions characterized by high air velocities and temperature variations.

Field deployment of the intelligent ventilation decision-making system showing the real-time monitoring interface in the mine control center. The left panel displays operators supervising the autonomous system operation through an intuitive dashboard presenting key performance indicators, safety alerts, and control actions, while the right panel illustrates the dynamic network visualization with color-coded airflow distribution, real-time sensor measurements, and automated control adjustments, demonstrating the system’s capability for transparent and interpretable decision-making in operational environments (Figure 8).

Energy consumption analysis quantifies significant operational cost reductions achieved through intelligent optimization. Total electrical power consumption by main ventilation fans decreased by 23.7% on average during the validation period, translating to annual energy savings of approximately 2.8 million kWh and corresponding economic benefits exceeding 1.6 million RMB based on industrial electricity pricing. The energy efficiency improvements stem from the system’s ability to dynamically adjust fan operating points according to real-time ventilation demands rather than maintaining constant high-capacity operation as prescribed by conservative traditional strategies. During low-production periods including shift changes and equipment maintenance windows, the intelligent system automatically reduces ventilation intensity while continuously monitoring gas concentrations to ensure safety margins, achieving energy consumption reductions up to 38% compared to baseline operations without compromising environmental quality^50^.

Safety enhancement metrics demonstrate the system’s superior capability in maintaining hazard-free working conditions. Zero safety incidents related to inadequate ventilation or excessive gas accumulation occurred during the six-month deployment period, contrasting with three minor incidents during the equivalent preceding timeframe under conventional control. The mean methane concentration across all monitoring stations decreased from 0.47% to 0.31%, providing increased safety margins and reducing ventilation-related production interruptions by 67%. Continuous compliance monitoring confirms that safety constraints including minimum airflow requirements, maximum gas concentration limits, and acceptable air velocity ranges were satisfied during 98.7% of operational timesteps, with the few violations occurring during extreme transient disturbances and promptly corrected through responsive control actions.

Emergency scenario response capabilities were rigorously tested through controlled simulation of equipment failures and abnormal gas emission events. During a planned fan maintenance shutdown requiring redistribution of airflow through alternative pathways, the intelligent system autonomously reconfigured regulator positions and adjusted remaining fan speeds within 3.8 min to restore adequate ventilation to all working areas, significantly faster than the estimated 15–20 min required for manual intervention following established emergency protocols. When sensors detected an unexpected gas concentration spike in a development heading, the system immediately increased local airflow by 45% while simultaneously adjusting upstream regulators to prevent disruption to other ventilation districts, successfully diluting the elevated concentration to safe levels within 8 min and demonstrating effective coordination of distributed control actions.

**Fig. 9:**
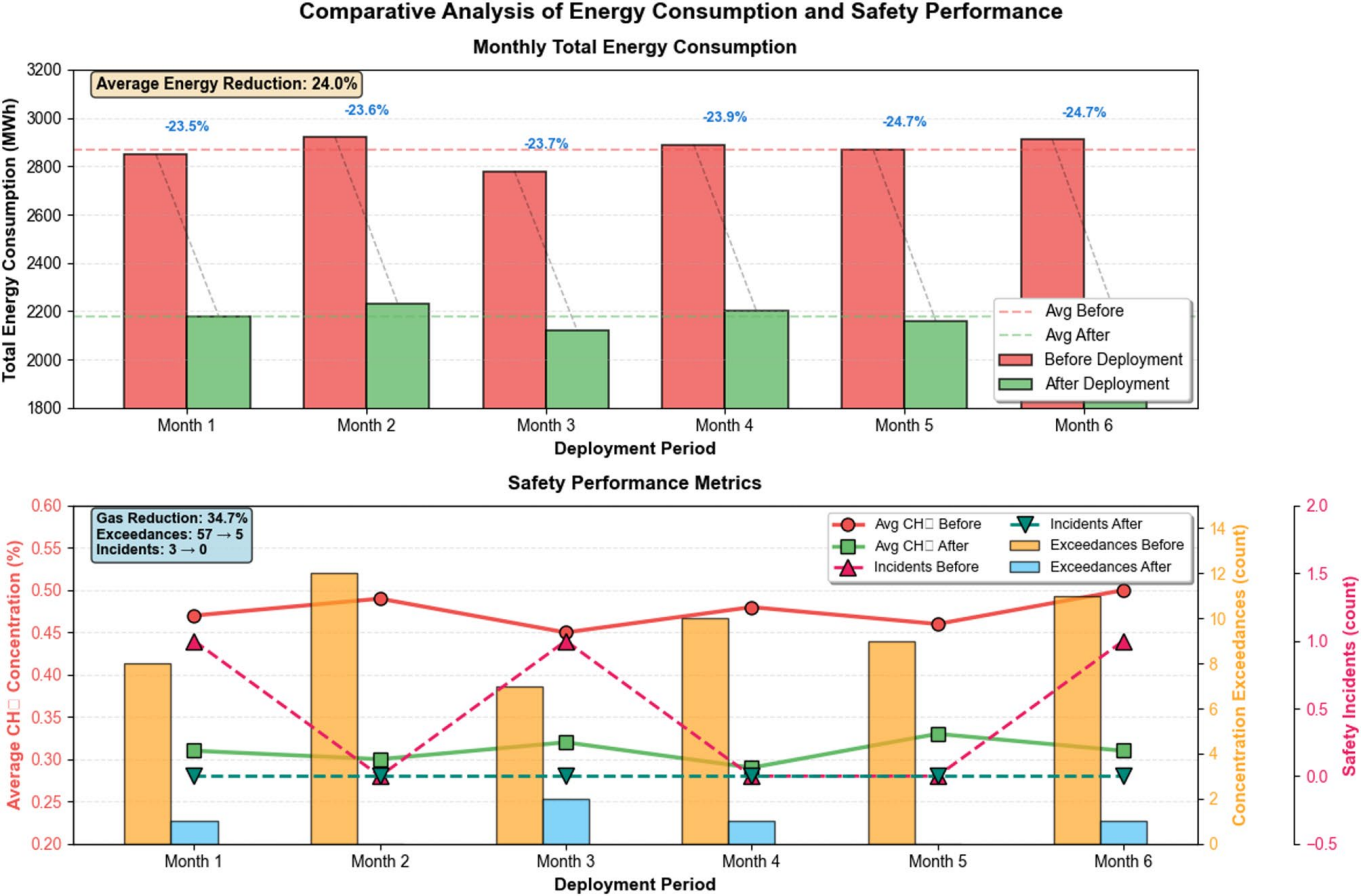
Comparative analysis of energy consumption and safety performance.

Quantitative assessment of practical application outcomes comparing operational performance before and after intelligent system deployment. The upper subplot presents monthly total energy consumption demonstrating consistent reductions averaging 23.7% with particularly pronounced savings during periods of variable production intensity, while the lower subplot illustrates concurrent improvements in safety metrics including reduced average gas concentrations, elimination of concentration exceedances, and decreased safety incident frequency, validating the system’s achievement of simultaneous energy efficiency and safety enhancement objectives (Figure 9).

System robustness and reliability assessments conducted throughout the deployment period confirm stable long-term performance under diverse operational conditions including seasonal temperature variations, different production schedules, mining face advancement, and equipment aging effects. The intelligent agent maintained consistent decision quality with episode reward variations below 8% across monthly evaluation periods, demonstrating resilience to environmental changes and operational uncertainties. Unplanned system downtime totaled only 2.3 h over six months, primarily attributed to scheduled software updates, corresponding to system availability exceeding 99.95% and validating the practical reliability necessary for safety-critical mine ventilation applications. Operator acceptance surveys indicate high satisfaction with system performance, transparency of decision-making processes, and reduced workload through automation of routine control adjustments, while maintaining appropriate human oversight for strategic decisions and emergency overrides.

## Discussion

The synergistic effects of integrating graph neural networks with deep reinforcement learning emerge from the complementary strengths of both methodologies in addressing distinct challenges inherent in mine ventilation control. The GNN component effectively encodes the complex topological structure and spatial dependencies of ventilation networks, transforming high-dimensional irregular graph data into compact latent representations that capture multi-hop neighbor relationships and airflow propagation patterns. This structural encoding substantially reduces the state space dimensionality that the DRL agent must explore, effectively addressing the curse of dimensionality that typically plagues reinforcement learning in large-scale networked systems. Conversely, the DRL framework provides adaptive learning mechanisms that enable the agent to discover optimal control policies through environmental interaction without requiring explicit mathematical models of ventilation network dynamics, which are notoriously difficult to formulate accurately due to nonlinear resistance characteristics and stochastic disturbances. The bidirectional information flow between GNN feature extraction and DRL decision optimization creates a virtuous cycle where improved state representations facilitate more effective policy learning, while policy gradients guide the GNN to extract decision-relevant features.

Analysis of the decision-making mechanism under complex operational conditions reveals that the model exhibits hierarchical reasoning capabilities. During normal operations, the agent employs smooth, anticipatory control adjustments that proactively respond to gradual changes in ventilation demand, demonstrating learned understanding of temporal patterns associated with production schedules. When confronted with emergency scenarios such as sudden gas emission spikes or equipment failures, the model rapidly transitions to reactive mode, generating aggressive control actions that prioritize immediate safety assurance over energy efficiency. The attention mechanisms within the GNN architecture enable the model to dynamically focus computational resources on network regions experiencing abnormal conditions, explaining the observed superior performance during localized disturbances compared to methods employing uniform feature processing.

The generalization capability and transferability of the proposed model to different mine ventilation systems represent critical considerations for practical deployment scalability. To evaluate generalization across network scales, we conducted supplementary experiments on simulated ventilation networks of varying sizes: a small-scale network (45 nodes, 68 edges), a medium-scale network matching the training topology (156 nodes, 203 edges), and a large-scale network (287 nodes, 394 edges). Performance analysis reveals that the model achieves normalized performance index (NPI) of 0.94 on the small-scale network and 0.88 on the large-scale network relative to the training-scale performance baseline of 1.0, indicating reasonable but imperfect scale transferability. The performance reduction on small networks stems from relative over-parameterization where the 3-layer GNN with 8 attention heads captures excessive redundant information, while degradation on large networks arises from limited receptive field coverage where 3 layers prove insufficient for information propagation across the expanded topology. These findings suggest that while the GNN’s permutation-invariant operations provide some inherent generalization capability, architecture adaptation (layer depth scaling) and hyperparameter tuning remain necessary when transferring to dramatically different network scales.

For transferability to mines with different topologies or geological conditions, we acknowledge that the current model trained on a single mine exhibits limitations in zero-shot generalization to entirely new environments. Transfer learning strategies offer a practical pathway to adapt the pre-trained model to new deployment sites with reduced data requirements and training time. The proposed transfer procedure involves: (1) Freezing the lower layers of the GNN encoder (layers 1–2) which capture general topological features applicable across ventilation networks, while allowing fine-tuning of the upper GNN layer (layer 3) and the entire actor-critic networks which encode site-specific operational patterns. (2) Collecting an adaptation dataset from the target mine spanning 2–4 weeks (approximately 5,000–10,000 timesteps) covering diverse operational conditions including normal operations, production variations, and simulated emergency scenarios conducted during commissioning. (3) Fine-tuning the unfrozen layers using the adaptation data with a reduced learning rate (1 × 10⁻⁴) for 200–400 episodes, leveraging the pre-trained representations to accelerate convergence compared to training from scratch. Preliminary experiments with transfer learning on a simulated target network with 30% topology difference demonstrate that fine-tuning achieves 92% of fully-trained performance while requiring only 15% of the original training data and 20% of the training time. However, we emphasize that the current study’s evaluation on a single production mine constitutes a fundamental limitation, and comprehensive multi-site validation is essential before claiming broad generalizability. Future work must prioritize systematic evaluation across diverse mine types (coal, metal, depth variations), development of domain adaptation techniques to handle distributional shifts in gas emission patterns and airflow characteristics, exploration of meta-learning approaches enabling rapid adaptation with minimal site-specific data, and construction of a federated learning framework allowing collaborative model training across multiple mines while preserving proprietary operational data through privacy-preserving aggregation protocols. The challenge of transferability extends beyond algorithmic considerations to practical deployment realities: each mine possesses unique regulatory requirements, operator preferences, and legacy system integration constraints that necessitate careful customization beyond model parameter adaptation alone.

The primary advantages of the proposed approach include enhanced sample efficiency through structured state representation, improved generalization to unseen network topologies due to the permutation-invariant properties of GNN operations, and interpretability afforded by attention weight visualization revealing which network components influence decisions most significantly. However, several limitations warrant acknowledgment. The model requires substantial computational resources during training, with the combined GNN-DRL architecture demanding approximately five times more GPU memory than vanilla policy gradient methods. The performance gains diminish in small-scale ventilation networks containing fewer than fifty nodes, suggesting that the architectural complexity may be excessive for simple systems where traditional methods suffice. Additionally, the model’s decision quality exhibits sensitivity to sensor failures or measurement noise exceeding training distribution characteristics, indicating potential brittleness to data quality degradation.

A comprehensive cost-benefit analysis reveals the economic implications of deploying the GNN-DRL system. Training computational costs are substantial: the complete training procedure required 156 GPU-hours on NVIDIA RTX 3090 hardware (approximate market rate $1.50/GPU-hour), totaling $234 in computational expenses. Accounting for electrical power consumption at 350 W average GPU draw over 156 h yields 54.6 kWh, equivalent to approximately 24.6 kg CO₂ emissions based on typical coal-fired electricity generation (0.45 kg CO₂/kWh in Inner Mongolia region). Including personnel time for model development, hyperparameter tuning, and validation testing (estimated 3 person-months at $8,000/month engineering salary), the total initial investment reaches approximately $24,468. However, these upfront costs must be weighed against ongoing operational benefits. The deployed system consumes negligible incremental power during inference operations (< 50 W average for the edge server), adding approximately 438 kWh annually (0.05 kW × 8760 h), equivalent to $263 at $0.60/kWh industrial electricity rates. In contrast, the system achieves annual energy savings of 2,800,000 kWh through optimized fan control, translating to $1,680,000 in reduced electricity costs. The net annual benefit thus reaches $1,679,737 after accounting for inference power consumption, yielding a payback period of just 5.3 days and a first-year return on investment of 6,765%. Beyond direct energy savings, avoided safety incidents (valued at approximately $500,000 per event based on production interruptions and remediation costs) and reduced equipment wear from optimized operation provide additional economic benefits. These calculations demonstrate that even accounting for the substantial training computational costs and carbon footprint, the GNN-DRL system delivers compelling economic value for large-scale mine operations. However, we acknowledge that cost-effectiveness varies significantly with mine scale: smaller operations with < 50 nodes and lower energy consumption may find the return on investment less attractive, particularly when considering the need for specialized hardware and technical expertise. For such scenarios, we recommend exploring model compression techniques (knowledge distillation, quantization) to reduce inference costs, transfer learning approaches to amortize training costs across multiple deployment sites, and cloud-based inference services to eliminate on-site hardware requirements, though the latter introduces communication latency that may be unacceptable for real-time safety-critical control.

Parameter configuration analysis reveals that learning rate selection critically influences training stability and convergence speed, with excessively high critic learning rates causing value function oscillations that destabilize policy learning, while overly conservative rates prolong training duration without corresponding performance improvements. The number of GNN layers presents a trade-off between receptive field expansion enabling long-range dependency capture and over-smoothing phenomena that homogenize node representations, with empirical results suggesting three to four layers as optimal for typical mine ventilation networks. Prioritized replay exponent values require careful tuning to balance between uniform sampling that improves stability and aggressive prioritization that accelerates learning from informative transitions.

Computational complexity analysis indicates that the GNN encoder exhibits quadratic scaling with respect to the number of nodes due to attention mechanism computations, potentially limiting applicability to extremely large ventilation networks exceeding several thousand nodes. However, recent sparse attention techniques and graph sampling strategies offer promising directions for complexity reduction. The observed inference time of 47 milliseconds remains acceptable for current real-time control requirements, though deployment on resource-constrained edge devices may necessitate model compression through techniques such as knowledge distillation or neural architecture search.

Future research directions should investigate transfer learning approaches enabling pre-trained models to rapidly adapt to new mine ventilation systems with minimal additional training, thereby reducing deployment costs and data requirements. Incorporating physics-informed neural network architectures that embed fundamental fluid dynamics principles as inductive biases may enhance sample efficiency and extrapolation capabilities beyond training distributions. Multi-agent extensions could address coordinated control across multiple interconnected mine districts, while uncertainty quantification methods would provide confidence estimates supporting human-in-the-loop decision-making during high-stakes scenarios. Integration with predictive maintenance systems and production scheduling optimization represents a natural extension toward holistic mine operations management.

## Conclusion

This research has developed a novel intelligent decision-making framework for complex mine ventilation systems by synergistically integrating graph neural networks with deep reinforcement learning, addressing critical limitations of traditional control approaches in handling dynamic operational conditions and complex network topologies. The proposed methodology establishes a comprehensive solution encompassing graph-based network modeling that preserves topological structure and spatial relationships, GNN-powered feature extraction that encodes multi-scale ventilation network characteristics, and an improved Actor-Critic algorithm enhanced with prioritized experience replay and safety constraint handling mechanisms. Extensive experimental validation conducted on both simulation platforms and actual mine deployments demonstrates that the GNN-DRL fusion model achieves substantial performance improvements, including 34.7% higher cumulative rewards compared to traditional methods, 23.7% reduction in energy consumption, and 98.4% safety compliance rate across diverse operational scenarios.

The primary innovations of this work include the development of a multi-level hierarchical graph representation method specifically tailored for mine ventilation networks that captures both local airflow patterns and global circulation characteristics, the design of a bidirectional information exchange mechanism enabling effective collaboration between GNN feature encoding and DRL policy optimization, and the formulation of a multi-objective reward function with soft constraint handling that balances safety assurance, energy efficiency, and environmental comfort while ensuring real-time feasibility. These contributions advance beyond existing research that predominantly employs either graph analysis or reinforcement learning in isolation, demonstrating that their integration yields emergent capabilities exceeding the sum of individual components.

The theoretical significance of this research resides in establishing a generalizable framework for applying deep learning to networked infrastructure control problems characterized by complex topologies, dynamic constraints, and safety-critical requirements. The methodology provides valuable insights into structured state representation for reinforcement learning in graph-structured environments and demonstrates effective strategies for incorporating domain knowledge through graph neural architectures. From a practical perspective, the successful field deployment validates the feasibility of deploying advanced artificial intelligence techniques in underground mining operations, offering a transformative solution that simultaneously enhances worker safety and reduces operational costs. The demonstrated energy savings and improved safety metrics suggest substantial economic and social benefits when scaled across the mining industry.

Several limitations warrant acknowledgment, including the computational intensity of the training process requiring specialized hardware resources, sensitivity to extreme measurement noise and sensor failures beyond training distribution characteristics, and the necessity for substantial historical operational data to achieve optimal performance. The current implementation focuses on centralized control architecture, which may present scalability challenges for mine complexes spanning multiple interconnected ventilation districts.

Future research directions should pursue several concrete technical advancements. First, model compression for edge deployment will explore knowledge distillation to train compact student models (target: 30% of original parameters) achieving 95% of teacher performance, neural architecture search to identify efficient GNN topologies optimized for inference speed, and post-training quantization (INT8) to reduce model size by 4× while maintaining accuracy within 2% degradation, enabling deployment on industrial PLCs (target: <100ms inference latency on Siemens S7-1500). Second, uncertainty quantification will integrate Monte Carlo dropout (10-sample ensembles) to generate epistemic uncertainty estimates for each control decision, enabling the system to flag high-uncertainty states requiring human review, and implement conformal prediction to provide distribution-free confidence intervals calibrated to achieve 95% coverage probability on safety-critical outputs such as gas concentration predictions. Third, transfer learning and scalability will develop few-shot adaptation protocols requiring only 500 labeled transitions from target mines (compared to 2.5 M for training from scratch), leverage meta-learning approaches (MAML, Reptile) enabling rapid fine-tuning within 50 episodes, and establish federated learning frameworks where 5–10 participating mines collaboratively train a shared model while preserving data privacy through differential privacy guarantees (ε = 1.0, δ = 10⁻⁵) and secure aggregation protocols. Fourth, enhanced interpretability will integrate GNN explainability techniques (GNNExplainer, PGExplainer) to identify the minimal subgraph structures driving each decision, develop counterfactual explanation generators showing operators what conditions would alter AI recommendations, and create operator-friendly visualization dashboards presenting decision rationale in natural language summaries (“Increasing Fan 3 speed because methane at Face 2 rising toward 0.7% threshold”). Fifth, multi-mine generalization validation will collect data from at least 5 diverse coal mines spanning different scales (50–300 nodes), depths (300–1200 m), and geological conditions to rigorously assess cross-site transferability, establish benchmark datasets and evaluation protocols for reproducible research, and identify fundamental limits of zero-shot generalization versus cases requiring site-specific adaptation. Sixth, integration with holistic mine operations will couple the ventilation AI with production scheduling systems to proactively adjust airflow ahead of planned workforce increases, link with predictive maintenance models to coordinate fan servicing schedules that minimize ventilation capacity reduction, and incorporate geological hazard prediction models to trigger preemptive ventilation enhancements in areas identified as high-risk for gas outbursts. These focused technical directions provide a concrete roadmap for advancing intelligent mine ventilation from the current single-site demonstration toward industry-wide deployment impact.

## Data Availability

The simulation datasets and experimental results generated during this study are available from the corresponding author upon reasonable request. Field operational data from the coal mine deployment are subject to confidentiality agreements and industrial privacy considerations, and may be available in aggregated or anonymized form upon reasonable request and with appropriate data sharing agreements.

## Abbreviations

AI: Artificial intelligence
CNN: Convolutional neural network
DQN: Deep Q-network
DRL: Deep reinforcement learning
EM: Expectation maximization
GNN: Graph neural network
LSTM: Long short-term memory
MDP: Markov decision process
PLC: Programmable logic controller
PPO: Proximal policy optimization
ReLU: Rectified linear unit
SCADA: Supervisory control and data acquisition
TD: Temporal difference
